# IL-8 as a urinary biomarker for the detection of bladder cancer

**DOI:** 10.1186/1471-2490-12-12

**Published:** 2012-05-04

**Authors:** Virginia Urquidi, Myron Chang, Yunfeng Dai, Jeongsoon Kim, Edward D Wolfson, Steve Goodison, Charles J Rosser

**Affiliations:** 1 Cancer Research Institute, MD Anderson Cancer Center Orlando, Orlando, FL, 32827, USA; 2 Department of Biostatistics, The University of Florida, Gainesville, FL, 32611, USA; 3 Department of Pharmacology, The University of Florida, Gainesville, FL, 32610, USA; 4 Section of Urologic Oncology, MD Anderson 391 Cancer Center Orlando, 1400 S. Orange Ave, Orlando, FL, 32806, USA

**Keywords:** IL-8, Biomarkers, Diagnosis, Bladder cancer

## Abstract

**Background:**

Current urine-based assays for bladder cancer (BCa) diagnosis lack accuracy, so the search for improved biomarkers continues. Through genomic and proteomic profiling of urine, we have identified a panel of biomarkers associated with the presence of BCa. In this study, we evaluated the utility of three of these biomarkers, interleukin 8 (IL-8), Matrix metallopeptidase 9 (MMP-9) and Syndecan in the diagnosis of BCa through urinalysis.

**Methods:**

Voided urines from 127 subjects, cancer subjects (n = 64), non-cancer subjects (n = 63) were analyzed. The protein concentrations of IL-8, MMP-9, and Syndecan were assessed by enzyme-linked immunosorbent assay (ELISA). Data were also compared to a commercial ELISA-based BCa detection assay (BTA-Trak©) and urinary cytology. We used the area under the curve of a receiver operating characteristic (AUROC) to compare the performance of each biomarker.

**Results:**

Urinary protein concentrations of IL-8, MMP-9 and BTA were significantly elevated in BCa subjects. Of the experimental markers compared to BTA-Trak©, IL-8 was the most prominent marker (AUC; 0.79; 95% confidence interval [CI], 0.72-0.86). Multivariate regression analysis revealed that only IL-8 (OR; 1.51; 95% CI, 1.16-1.97, *p* = 0.002) was an independent factor for the detection of BCa.

**Conclusions:**

These results suggest that the measurement of IL-8 in voided urinary samples may have utility for urine-based detection of BCa. These findings need to be confirmed in a larger, prospective cohort.

## Background

Voided urinary cytology (VUC) remains the most established adjunct to cystoscopy in the detection of BCa. While VUC has a specificity of >93%, its sensitivity is only 25-40%, especially for low-grade and low-stage tumors [1]. Commercial tests measuring nuclear matrix protein (NMP-22) and bladder tumor antigen (BTA) have emerged as diagnostic urinary protein assays for BCa. The analysis of naturally shed urothelial cells isolated from the urine, as used in VUC, can be enhanced by combining VUC with molecular assays to detect protein biomarkers or aberrant chromosomes. ImmunoCyt© uses fluorescent monoclonal antibodies to detect mucin antigen and carcinoembryonic antigen on urothelial cells [2]. Urovysion©, a fluorescence *in situ* hybridization assay designed to detect aneuploidy for chromosomes 3, 7, 17, and loss of the 9p21, can be applied to VUC slides [3]. Unlike VUC, these tests have a lower specificity, thus there is currently no urine test for BCa that dominates the field.

The inadequate power of a single biomarker may partly explain why detecting BCa using urinalysis remains a challenge. There needs to be an evolution towards tests that monitor multiple biomarkers in order to achieve the desired diagnostic accuracy. We have recently identified diagnostic molecular signatures for BCa using a number of discovery approaches. Utilizing genome-wide mRNA profiling, we analyzed shed urothelia isolated from 92 voided urine samples. Microarray analysis identified the expression of 52 genes as being significantly associated with BCa. The expression of a selection of this geneset was subsequently validated by quantitative PCR in an independent cohort of 81 patients (manuscript submitted). In a separate study, we utilized a dual-lectin affinity chromatography and mass spectrometry platform to analyze the supernatant of voided urine samples from 100 subjects (54 cancer subjects and 46 non-cancer subjects). The analyses identified 265 distinct glycoproteins present in urine, and several that were significantly associated with the presence of BCa. A panel of 9 glycoproteins was subsequently validated by protein assay [4]. Next the above genomics and proteomics data were integrated and analyzed by sophisticated bioinformatics leading to a panel of candidate biomarkers. Our ultimate aim is to validate the panel of candidate biomarkers in patients with newly diagnosed BCa. In this study, we investigated the utility of three of the potential BCa candidate biomarkers, interleukin 8 (IL-8), matrix metallopeptidase 9 (MMP-9) and Syndecan, by measuring the presence of these proteins in voided urine samples obtained from an independent cohort of 127 subjects.

## Methods

### Specimen and data collection

Under Institutional Review Board approval and informed consent (IRB #560-2006), voided urine samples, and associated clinical information were prospectively collected. The study cohort consisted of 63 individuals with no previous history of urothelia carcinoma, gross hematuria, active urinary tract infection or urolithiasis, and 64 individuals with newly diagnosed primary urothelia cell carcinoma. Median follow-up was 11.5 months. This cohort of 127 subjects served as our phase II (validation study) according to the International Consensus Panel on Bladder Tumor Markers [5] and was reported based on the STARD criteria [6]. All subjects were evaluated in the outpatient Urology clinic. Urinalysis, urinary cytology and BTA-Trak© were performed on all subjects. Furthermore, in our cancer group, axial imaging of the abdomen and pelvis and cystoscopy were performed, and urothelial cell carcinoma was confirmed by histological examination of excised tissue. Pertinent information on clinical presentation, staging and histologic grading [7,8] and outcome were recorded (Table 1).

**Table 1:** Demographic, clinicopathologic characteristics and concentration of urinary proteins in the study cohort

	**Non-cancer (%)**	**Cancer (%)**
	N=63	N=64
Median Age (range, y)	60 (30-81)	69.5 (22-90)
Male : Female ratio	55 : 8	55 : 9
**Race**		
White	41 (65)	58(91)
African American	8 (13)	0(0)
Other	14 (22)	6 (9)
Tobacco use	25 (40)	54 (84)
Gross hematuria	0 (0)	47 (73)
Suspicious/positive cytology	1 (2)	18 (28)
Median follow-up (months)	11.5	12.0
**Clinical stage**		
Tis^	n/a	6(9)
Ta	n/a	15(23)
T1	n/a	9 (14)
T2	n/a	31(48)
T3	n/a	4 (6)
T4	n/a	2 (3)
N+ ~	n/a	3 (5)
**Grade**		
Low	n/a	9(14)
High	n/a	55(86)
**Urinary Proteins**	Median (range)	Median (range)
IL-8 (pg/ml)	0(0 - 134.33)	128.43 (0 - 17140.2)
MMP–9 (ng/ml)	0 (0 – 14.25)	0.95 (0 – 1002.6)
Syndecan (ng/ml)	40.67(0 - 199.55)	31.81(0 - 335.18)
BTA-Trak (U/ml)	13.13(0.5 - 36.87)	179.33 (0 - 24865.4)
Hemoglobin (ng/ml)	0(0 - 125.92)	8.73 (0 - 130367.5)
	Mean + SD	Mean + SD
IL-8 (pg/ml)	05.02 ± 20.99	1335.07 ±3495.26
MMP–9 (ng/ml)	0.35 ±1.83	46.76 ±142.16
Syndecan (ng/ml)	49.10 ± 42.09	55.04 ± 74.88
BTA-Trak (U/ml)	14.66 ± 8.05	1630.5 ± 3954.5
Hemoglobin (ng/ml)	2.63±15.97	4451.3±22814.4

^, 4 subjects with concomitant cis had T1 (n=2) and T2 (n=2) disease.

~, Subjects with T2 (n=1), T3 (=1) and T4 (n=1) disease and node positive.

### Specimen processing and analysis

Prior to any type of therapeutic intervention, 100 mL of voided urine was obtained from each subject. Fifty milliliters of urine was sent to the clinical laboratory for urinalysis and urinary cytology. The remaining 50 ml of urine was assigned a unique identifying number before immediate delivery and laboratory processing. Each urine sample was centrifuged at 600 × *g* 4°C for 5 min. The supernatant was decanted and aliquoted, and the urinary pellet was snap frozen. Both the supernatant and pellet were stored at -80 ° C prior to analysis. Urine supernatant protein concentration was determined using Pierce 660-nm Protein Assay Kit (Thermo Fisher Scientific Inc., Waltham, MA, USA).

Enzyme-linked Immunosorbent Assays for IL-8, MMP-9, Syndecan, Hemoglobin and Bladder Tumor Antigen (BTA).

The levels of human IL-8 (Cat # ab46032 Abcam, Cambridge, MA, USA), human MMP-9 (Cat# DMP900 R&D Systems Inc., Minneapolis, MN, USA) and human Syndecan (Cat# ab46507 Abcam, Cambridge, MA, USA) were monitored in urine samples using enzyme-linked immunosorbent assays. Commercially available ELISA assays were used to measure levels of urinary hemoglobin (Cat#E88-135 Bethyl Laboratories Inc., Montgomery, TX, USA) and BTA (BTA-Trak© Ca# 662150 Polymedco Inc. Cortlandt Manor, NY, USA). The assays were conducted according to the manufacturer’s instructions. Laboratory personnel were blinded to final diagnosis. Calibration curves were prepared using purified standards for each protein assessed. Curve fitting was accomplished by either linear or four-parameter logistic regression following manufacturer’s instructions. Protein concentration levels were normalized using urinary creatinine concentrations.

### Data analysis

The association between a biomarker and BCa was tested using Wilcoxon rank sum test. Spearman rank correlation coefficients were used to examine the correlation between urinary tumor biomarker (IL-8, MMP-9, Syndecan and BTA) concentrations and urinary hemoglobin concentration. Nonparametric receiver operating characteristic (ROC) curves were generated in which the value for sensitivity is plotted against false-positive rate (1-specificity). Areas under ROC curves were estimated and compared by chi-square test. We defined a diagnostic test (positive vs. negative) for BCa using a cutoff threshold for each biomarker. The optimal cutoff (Youden index) was selected to maximize the sum of the sensitivity and specificity [9]. The overall accuracy of a biomarker to predict BCa is defined as the average of the sensitivity and the specificity. To assess the independent association between biomarkers, logistic regression analysis with BCa status (yes vs. no) as the dependent variable and IL-8, MMP-9, Syndecan, BTA concentrations as explanatory variables. In multivariate logistic analysis, the log-transformation was used to reduce the skewness of each biomarker. Statistical significance in this study was set at *p* < 0.05 and all reported *p* values were 2-sided. All analyses were performed with SAS software version 9. 3.

## Results

**Figure 1:**
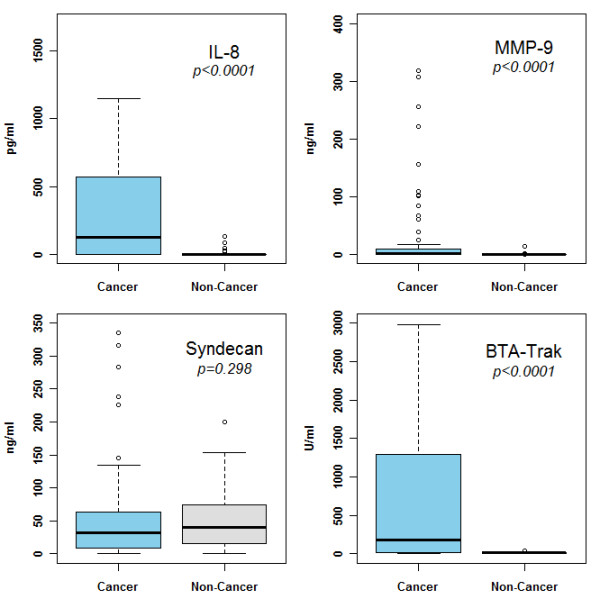
**Comparison of urine concentrations of interleukin 8 (IL-8), matrix metallopeptidase 9 (MMP-9), Syndecan and bladder tumor antigen (BTA) between the cancer and non-cancer groups.** Median levels are depicted by horizontal lines. Significance (*p < 0.05*) was assessed by the Wilcoxon rank sum test.

The cohort of 127 subjects consisted of 64 subjects with active BCa and 63 control subjects. Demographic, clinical and pathologic characteristics of both groups are illustrated in Table 1. In the cancer cohort, urinary cytology only had a sensitivity of 28%. Median urinary concentrations of IL-8 (128.43 pg/ml vs. 0 pg/ml, *p* < 0.0001), MMP-9 (0.95 ng/ml vs. 0 ng/ml, *p* < 0.0001), and BTA (179.34 U/ml vs. 13.13 U/ml, *p* < 0.0001) were significantly higher in subjects with BCa compared to subjects without BCa. Median urinary Syndecan concentration in the cohort with BCa was similar to that in the cohort without BCa (31.81 ng/ml vs. 40.67 ng/ml, *p* = 0.30). ELISA data are presented in a boxplot figure (Figure 1).

The level of hematuria was assessed in all cases by reviewing the clinical record for each subject, by analyzing voided urine samples with Clinitek point of care urinalysis testing©, and quantitatively by performing a hemoglobin ELISA on all urine samples. The qualitative analyses revealed that 83% of BCa subjects were noted to have hemoglobin in their urine samples compared with only 23% of control subjects. The median urinary levels of hemoglobin in BCa subjects compared to control subjects were 8.73 ng/ml vs. 0 ng/ml, *p* < 0.0001. Interestingly, of the four biomarkers assessed, BTA had the highest correlation coefficient to urinary hemoglobin (0.732).

**Figure 2:**
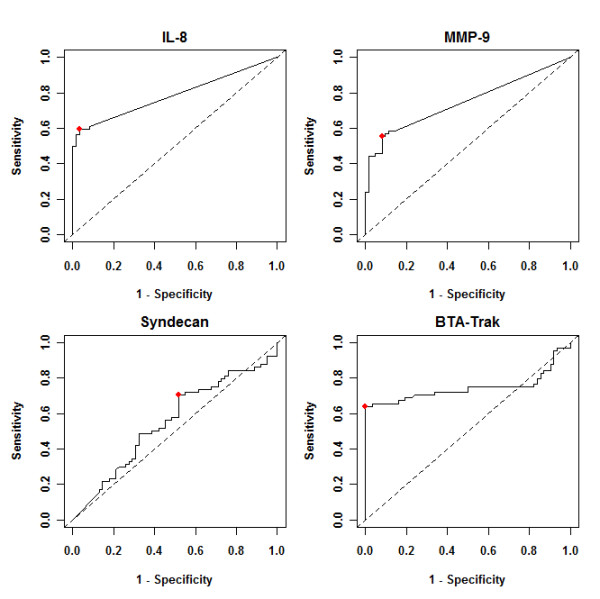
**(a) Receiver operating characteristic (ROC) curves for urinary IL-8, MMP-9, Syndecan and BTA.** Based on the area under the ROC curve (AUROC), Youden Index cutoff values that maximized the sum of sensitivity and specificity were determined for each biomarker (crossed square on curve). Table provides performance values for each biomarker. PPV, positive predictive value. NPV, negative predictive value. **(b)** Receiver operating characteristic (ROC) curves for the combination of IL-8, MMP-9 and Syndecan. Based on the area under the ROC curve (AUROC), Youden Index cutoff values that maximized the sum of sensitivity and specificity were determined for combination of IL-8, MMP-9 and Syndecan. Table provides performance values for each biomarker. PPV, positive predictive value. NPV, negative predictive value.

The ability of the above biomarkers to predict the presence of BCa was analyzed using nonparametric ROC analyses and the area under the ROC curve (AUROC), according to National Cancer Institute guidelines [10]. We determined the Youden Index cutoff values to maximize the sum of sensitivity and specificity. BTA served as our positive control and was noted to have an AUROC of 0.75 (95% CI: 0.66-0.85, Figure 2). Using the Youden Index cutoff value (Figure 2), urinary BTA provided a sensitivity of 64%, specificity of 100%, positive predictive value of 100%, negative predictive value of 73%, and an overall accuracy of 82%. Urinary IL-8 analyses (AUROC 0.79) had a sensitivity of 59%, specificity of 97%, positive predictive value of 95%, negative predictive value of 70%, and an accuracy of 78%. The difference in AUROC between IL-8 and BTA was not significant (0.79 vs. 0.75, p = 0.37). Urinary MMP-9 was also a relatively accurate biomarker for BCa detection (AUROC: 0.75; 95% CI: 0.68-0.82, Figure 2). Urinary MMP-9 analyses revealed a sensitivity of 56%, specificity of 92%, positive predictive value of 88%, negative predictive value of 67%, and an accuracy of 74%. Urinary Syndecan was not predictive for the presence of BCa (area under the curve: 0.55; 95% CI: 0.45-0.65, Figure 2). Urinary Syndecan monitoring resulted in a sensitivity of 70%, specificity of 48%, positive predictive value of 58%, negative predictive value of 61%, and an accuracy of 59%.

In multivariate logistic regression analysis, only elevated urinary IL-8 (OR: 1.51; 95% CI: 1.16-1.97; *p* = 0.002) was significantly associated with BCa. BTA, MMP-9 and Syndecan levels were not significantly associated with BCa in multivariate logistic regression analysis (Table 2).

**Table 2:** Logistic regression analysis of biomarkers in voided urine

**Variable**	**Coefficient**	**Odds Ratio**	**95% C.I.**	***p*-value**
IL-8	0.414	1.513	1.163 – 1.968	0.002
MMP-9	0.487	1.627	0.623 – 4.247	0.320
Syndecan	-0.152	0.859	0.642 – 1.149	0.306
BTA-Trak	0.241	1.272	0.852 – 1.900	0.240

## Discussion

Cancer of the urinary bladder is among the five most common malignancies worldwide. Urothelial cell carcinomas constitute approximately 95% of all bladder cancer cases, [11] and associated with tobacco exposure [12]. At presentation, more than 80% of bladder tumors are non-muscle invasive papillary tumors (Ta or T1), which harbor a 5-year survival rate of approximately 94%, [13] however, approximately 70% of patients with these lesions develop tumor recurrence within two years of initial diagnosis. The recurrence phenomenon of non-muscle invasive BCa makes it one of the most prevalent cancers world-wide (in America it is second only to colorectal cancer) and is, therefore, a great burden to healthcare systems [13]. Once BCa is detected and treated, patients will routinely get frequent surveillance cystoscopy to monitor for tumor recurrence [14]. If left untreated these initially non-invasive tumors can progress to muscle-invasive tumors which have significantly reduced 5-year survival rate [15]. Thus, early detection, ideally through non-invasive urine-based analysis, remains one of the most urgent issues in BCa research.

In the current report, we describe the analysis of IL-8, MMP-9 and Syndecan in a validation phase cohort of 127 subjects using ELISA assays. Urinary protein concentrations of IL-8, MMP-9 and BTA were significantly associated with BCa. IL-8 outperformed the other experimental biomarkers (specificity 97% and positive predictive value 95%). Multivariate logistic regression analysis highlighted only IL-8’s (OR: 1.51; 95% CI: 1.16-1.97) association with BCa.

In performing studies discovering urinary biomarkers, frozen (banked) urinary samples are critical. In the current study, the cellular component of voided urine was removed from the supernatant to prevent cellular lysis, which may skew results upon thawing analyzing. Upon reviewing available commercial assays to detect BCa in urine samples, only BTA has been confirm on frozen banked samples (see product insert) and thus BTA-Trak served as one of our controls. BTA-Trak© was the initial assay that was used to measure BTA in the urines of BCa subjects in a large multicenter trial [16]. Subsequently, BTA-Trak© was modified and incorporated into a point of care assay (BTA-stat©) which received FDA approval in 1997 and has become a standard tool in the non-invasive diagnosis ofBCa. BTA-Trak©, median sensitivity is quoted as 71% (range: 60–83%) with improved sensitivity in high-grade tumors. Median specificity is 66% (range: 60–79%), significantly lower than VUC [17].

Previous reports have implicated both IL-8 in bladder tumor biology and for use as biomarkers of BCa. IL-8 is an angiogenic factor associated with inflammation and carcinogenesis, and previous reports have documented elevated urinary protein levels of IL-8 in subjects with urothelial cell carcinoma [18-20]. Studies have indicated that elevated urinary levels are associated with increased stage of disease, [18] disease recurrence [19] and lack of efficacy of intravesical therapies, including bacillus Calmette-Guérin and mitomycin C [21]. As an initial diagnostic indicator, urinary IL-8 achieved a sensitivity of 59%, and a specificity of 90% in a study of 140 subjects [18] and 50%/90% in a study of 79 subjects, [20] results very much in line with our findings. In biological studies, IL-8 has been shown to have mitogenic and angiogenic properties, and high levels result in increased tumorigenicity, progression and metastasis in mouse models. Inhibition of tumor growth in mouse xenograft models by anti-IL-8 antibodies was shown to act via down-regulation of nuclear factor kappa-B [22].

Matrix metalloproteinase 9 (MMP-9) has been associated with tumor cell invasion and metastasis in many human cancers, including BCa. As a marker for BCa, studies have reported that elevated urinary protein levels of MMP-9 are associated with cancer. In a study of 188 subjects, high MMP-9 levels were significantly correlated with large tumor size and poor malignancy grade, and increasing levels were associated with poor overall survival [23]. Studies that measured urinary MMP-2 and MMP-9 by both ELISA and zymography have suggested that MMP-9 levels may be useful as an adjunct to cytology, [24] or as a diagnostic with good sensitivity (80%) [25]. Though we demonstrated that urinary protein MMP-9 levels were significantly elevated in BCa subjects by univariate analysis, this did not hold true in logistic regression analysis, and the sensitivity in our cohort was only 56%.

Similar to the study by Aaboe *et al.,*[26] we previously identified Syndecan, a transmembrane heparin sulfate proteoglycan, as a potential biomarker using gene expression profiling [27]. We went on to confirm that it was more highly expressed in urine samples from BCa subjects via quantitative PCR of shed urothelia cells, however, we were unable to confirm that Syndecan protein was significantly elevated in the urines from BCa subjects. This may be due to the fact that Syndecan is membrane-bound and so is less likely to be present in the soluble fraction of the urine, but a study has shown elevated serum Syndecan levels to be an independent prognostic marker for multiple myeloma [28].

In order to monitor whether experimental biomarkers were associated with hematuria, urinary hemoglobin levels were quantitated in each sample using ELISA. As expected, hemoglobin was revealed to be significantly elevated in the urines of subjects with BCa. Interestingly, the levels of urinary BTA had the highest correlation with urinary hemoglobin, raising the possibility that an appreciable source of BTA is the blood routinely found in BCa patients’ urine. This was previously suggested by Oge *et al.*[29] We are currently investigating this possibility in additional experiments. The accurate quantitation of hematuria using a hemoglobin ELISA provides valuable insight into the potential clinical utility of a biomarker prior to development of new assays designed to detect BCa in voided urine samples [30].

We recognize that our study has several limitations. First, processed, banked urines were analyzed. Urines were centrifuged and separated into cellular pellet and supernatant prior to storage at -80^0^ C. It is feasible that freshly voided urine samples may provide different results, and it is fresh urine that would be the material used for point-of-care assays. We are currently investigating the performance of selected biomarkers in urines processed via a number of different protocols. Second, it is uncertain how the protein composition of the urine supernatant may change during frozen storage. The number of freeze-thaw cycles was kept to 1–2 by dividing the urine supernatant into multiple small aliquots. Next, we are a tertiary care facility that is preferentially referred high grade, higher stage disease, which is reflected in our cohort. To confirm the robustness, subsequent studies will assess more urines from subjects with low-grade, low-stage disease in community urologic practices. Subsequently, the sensitivity of VUC in our cohort of predominantly high-grade (grade 3) disease (28%) was lower than would be expected. This calls into question the known inter-observer variability of interpreting VUC. In subsequent studies, we will utilize two cytopathologists to interpret these results. Lastly, our cohort was comprised of two cohorts: active cancer or control cases with no active cancer, no history of cancer, no urinary tract infection, no urolithiasis, and no gross hematuria. This may account for favorable detection levels with IL-8. Currently, we are assessing our panel of validated biomarkers in a large diverse cohort to further validate the robustness of this diagnostic signature.

## Conclusions

We have demonstrated that urinary levels of IL-8 can be indicative of BCa. The use of IL-8 in conjunction with other validated biomarkers from our global profiling may prove more efficacious than any single biomarker alone. Larger, prospective studies are needed to determine the potential utility of urinary IL-8 as a biomarker in the non-invasive evaluation of patients who are at risk of harboring BCa.

## Abbreviations

BCa, Bladder cancer; IL-8, Interleukin 8; MMP-9, Matrix metallopeptidase 9; ELISA, Enzyme-linked immunosorbent assay; BTA, Bladder tumor antigen; VUC, Voided urinary cytology; AUROC, Area under the curve of a receiver operating characteristic; ROC, Receiver operating characteristics.

## Competing interests

The authors declare that they have no competing interests.

## Authors’ contributions

All authors have read and approved the final manuscript. VU Study concept and design, drafting of manuscript, MC Statistical analysis, YD Statistical analysis, JK Acquisition of data, EDW Specimen collection and processing, SG Study concept and design, drafting of manuscript, supervision, CJR Study concept and design, drafting of manuscript, supervision.

## Pre-publication history

The pre-publication history for this paper can be accessed here:

http://www.biomedcentral.com/1471-2490/12/12/prepub
